# Vildagliptin has the same safety profile as a sulfonylurea on bone metabolism and bone mineral density in post-menopausal women with type 2 diabetes: a randomized controlled trial

**DOI:** 10.1186/s13098-017-0232-2

**Published:** 2017-05-15

**Authors:** Andre Gustavo Daher Vianna, Claudio Silva de Lacerda, Luciana Muniz Pechmann, Michelle Garcia Polesel, Emerson Cestari Marino, Victoria Zeghbi Cochenski Borba, Fellype de Carvalho Barreto

**Affiliations:** 1 Pontifical Catholic University of Parana, Rua Imaculada Conceição, 1155-Bloco Medicina-Prado Velho, Curitiba, Parana Zip code: 80215-901 Brazil; 2 Curitiba Diabetes Center, Division of Endocrinology, Hospital Nossa Senhora das Graças, Rua Alcides Munhoz, 433-4° andar-Mercês, Curitiba, Paraná Zip code: 80810-040 Brazil; 3 Division of Endocrinology, Department of Internal Medicine, Federal University of Paraná, Avenida Agostinho Leão Junior, 285-Alto da Gloria, Curitiba, Paraná Zip code: 80030-110 Brazil; 4 Division of Nephrology, Department of Internal Medicine, Federal University of Paraná, Rua General Carneiro, 181-Alto da Gloria, Curitiba, Paraná Zip code: 80060-900 Brazil

**Keywords:** Vildagliptin, Gliclazide MR, Postmenopausal, Type 2 diabetes, Bone turnover markers, Bone mineral density

## Abstract

**Background:**

Several antidiabetic therapies affect bone metabolism. Sulfonylureas have the lowest impact on bone among oral antidiabetics. The objective of this study is to compare the effects of vildagliptin and gliclazide modified release (MR) on bone turnover markers (BTMs) and bone mineral density (BMD) in postmenopausal women with uncontrolled type 2 diabetes (T2D).

**Methods:**

Forty-two postmenopausal women with uncontrolled T2D were randomly allocated into vildagliptin or gliclazide MR (control) groups. The primary endpoint was the change in the BTMs in months 6 and 12 compared with the baseline. The secondary endpoint was the variation in the BMD, which was assessed via dual-energy X-ray absorptiometry at the lumbar spine, femoral neck and total hip at baseline and month 12.

**Results:**

After a 12-month treatment, the BTM serum carboxy-terminal telopeptide of type 1 collagen increased 0.001 ± 0.153 ng/mL in the vildagliptin group versus 0.008 ± 0.060 ng/mL in the gliclazide MR group (*p* = 0.858). The serum osteocalcin, serum amino-terminal propeptide of procollagen type I and urinary amino-terminal telopeptide of type 1 collagen remained stable in both groups, and there was no statistically significant difference between the effect of vildagliptin and gliclazide MR on these variables. The lumbar spine BMD did not change in the vildagliptin or gliclazide MR groups after a 12-month treatment (0.000 ± 0.025 g/cm^2^ versus −0.008 ± 0.036, respectively, *p* = 0.434). Furthermore, there was a similar lack of change in the femoral neck and total hip BMD values in both treatments.

**Conclusions:**

Bone turnover markers and BMD remained unchanged after a 12-month treatment in both groups, which suggests that vildagliptin has the same safety profile as gliclazide MR on bone metabolism.

*Trial Registration* ClinicalTrials.gov number NCT01679899

**Electronic supplementary material:**

The online version of this article (doi:10.1186/s13098-017-0232-2) contains supplementary material, which is available to authorized users.

## Background

Increasing evidence suggests that type 2 diabetes (T2D) is an independent risk factor for fragility fractures, and the skeleton was recently recognized as another target tissue subject to diabetic complications [1]. The superposition of the molecular mechanisms that control bone homeostasis and energy metabolism creates a possibility in which T2D and antihyperglycemic therapies may similarly affect the skeleton [2, 3].

In this context, it has been postulated that dipeptidyl peptidase-4 inhibitors (iDPP-4) may produce positive effects on bone as a result of the regulation of various intestinal hormones, such as glucose-dependent insulinotropic peptide (GIP) and glucagon-like peptide-2 (GLP-2), which increase bone formation and prevent bone resorption [4–10]. Female mice treated with sitagliptin, an iDPP-4, exhibited significant improvements in the vertebral bone mineral density (BMD) and trabecular architecture [11]. A meta-analysis that compared 11,880 iDPP4 users with 9175 patients from the control group (placebo or active medications) suggested that iDPP4 may be associated with a 40% reduction in the risk of bone fractures [12].

Sulfonylureas likely have the lowest impact on bone metabolism among oral antidiabetics [2]. Gliclazide modified release (MR) is a highly potent and selective sulfonylurea that is considered a reference drug within this class because of its low risk of hypoglycemia compared with other sulfonylureas and its lack of increase in cardiovascular mortality [13, 14]. To date, there are no prospective randomized studies in humans that have examined the effect of the iDPP-4 vildagliptin with other classes of oral anti-diabetic agents on BMD and biochemical markers of bone remodeling, particularly in postmenopausal women. The comparison of vildagliptin with a sulfonylurea, seemingly neutral for bone metabolism, appears to be a useful approach to investigate the hypothesis that the former medication is also harmless for bone. This study was therefore designed to assess and compare the effects of a 12-month treatment of vildagliptin and gliclazide MR on bone metabolism and BMD in postmenopausal women with uncontrolled T2D.

## Methods

### Patients and study design

This study is part of the BoneGLIC study (registered at Clinical Trials.gov with Number NCT01679899). It is a 12-month, one-center (Curitiba Diabetes Center, Brazil), randomized controlled trial. All study subjects were postmenopausal women diagnosed with T2D treated with metformin, who fulfilled the following inclusion criteria: age ≥40 years old and glycated hemoglobin (HbA1c) ≥6.5% (48 mmol/mol) at randomization. The exclusion criteria included an acute cardiovascular event (cardiac, cerebral or peripheral), chronic dialysis and/or renal transplantation, serum creatinine >1.5 mg/dL, human immunodeficiency virus infection, severe autoimmune disease, chronic treatment with oral steroids (>30 consecutive days), current or previous treatment with incretin mimetics (iDPP-4 or GLP-1 receptor agonists), current or previous treatment with pioglitazone or rosiglitazone, body mass index (BMI) >50 kg/m^2^, HbA1c ≥9%, chronic or alcoholic liver disease, plasma triglycerides >1000 mg/dL (11.3 mmol/L), serum 25-hidroxi-vitamin D (25-OH-vit.D) <20 ng/mL, abnormal levels of parathyroid hormone (PTH), serum cortisol, insulin-like growth factor 1 (IGF-1) or growth hormone (GH) and history of previous fragility fracture. Patients were recruited via media outlets, including online and newspaper advertisements, flyers, radio announcements or after a search for T2D treatment at the research center.

The study protocol was approved by the site ethical review committee in agreement with the Declaration of Helsinki. All participating patients provided their written informed consent prior to screening.

Fifty-six postmenopausal women with T2D were enrolled from October 2012 to October 2014. Randomization occurred through the free online software Research Randomizer version 4.0 (https://www.randomizer.org/), which randomly generates numbers that indicate the specific group to which the research subject will be allocated [15]. Randomization and enrollment were performed by the study coordinator, and the medical investigators assigned participants to the intervention. Blinding was implemented when assessing outcomes, in which the staff personal who analyzed the outcomes did not have access to the identification of the patient examined or the group that the individual was allocated.

Vildagliptin (Novartis Pharma AG, Basel, Switzerland) was administered at 50 mg orally twice daily, and the comparator (control) gliclazide MR (Laboratoires Servier, Neuilly sur-Seine, France) was initially administered at 120 mg orally once daily (maximum dose). If the maximum dose of gliclazide MR was not tolerated or was otherwise associated with unacceptable adverse events, a dose reduction to 60 mg once daily was allowed at the discretion of the investigator. After randomization, the treatment with vildagliptin or gliclazide MR was open-label throughout the study. All patients in both groups received a similar orientation for diet and physical activity performed by the same study site nutritionist, with reinforcement throughout the study.

Coincidentally, all subjects randomized were treated with the oral antidiabetic metformin, and drug naïve or insulin-treated subjects were not randomized.

### Measurements

Anthropometric data were recorded at baseline and each study visit by the same investigator.

The primary study outcomes, the biochemical markers of bone turnover, serum carboxy-terminal telopeptide of type 1 collagen (CTX), serum osteocalcin (OC), serum amino-terminal propeptide of procollagen type I (PINP) and urinary amino-terminal telopeptide of type 1 collagen (U-NTX), were measured at baseline and month 6. Samples of U-NTX, CTX, and OC markers were also collected in month 12.

The secondary outcomes included the BMD of the lumbar spine, femoral neck and total hip that were measured at baseline and month 12. The safety secondary variables alanine aminotransferase (ALT) and aspartate aminotransferase (AST) were collected at baseline, months 6 and 12, and calcitonin was assessed at baseline and month 12.

Exploratory variables, including fasting plasma glucose (FPG), postprandial glucose (PPG) and glycated hemoglobin (HbA1c), were assessed at baseline and every three months until the end of the study at month 12.

Venous blood samples were collected between 7:30 and 9:00 a.m. in the fasting state to measure FPG, HbA1c, lipids, ALT, AST, bone formation markers OC, PINP and the bone resorption marker CTX. Samples were left to clot for 30 min and subsequently centrifuged. The serum was frozen and maintained at –70 °C and then unfrozen for the evaluation of bone turnover markers. PPG was collected 2 h after lunch. The second-morning void urine sample was collected to evaluate the bone resorption marker U-NTX (2 mL urine in a sterile screw cap container), and the results were reported in nmol of bone collagen equivalents per mmol creatinine (nmol BCE/mmol creatinine).

FPG and PPG were measured using the hexokinase method. HbA1c was measured via high-pressure liquid chromatography (HPLC). OC, CTX, and PINP were assessed using an electrochemiluminescence assay (Roche Diagnostics, Basel, Switzerland). The OC inter-assay and intra-assay variabilities are 3.2 and 1.2%, respectively (normal range 15.0–46.0 ng/mL for postmenopausal women). The CTX inter-assay and intra-assay variabilities are 7.8 and 3.9%, respectively (normal range 0.106–1.008 ng/mL for postmenopausal women). The PINP inter-assay and intra-assay variabilities are 2.0 and 1.6%, respectively (normal range 16.3–73.9 µg/L for postmenopausal women). U-NTX were measured via enzyme immunoassay (EIA, Grifols Asia Pacific Pte. Ltd, Singapore). The inter-assay and intra-assay variabilities are 17.2 and 8.6%, respectively (normal range 26.0–83.0 nmol BCE/mmol creatinine for postmenopausal women). The BMD was assessed in agreement with the standards of the International Society for Clinical Densitometry (ISCD) via dual-energy X-ray absorptiometry (DXA, Discovery W, Hologic Inc., Marlborough, MA, USA). The short-term in vivo precision error (root-mean-square standard deviation) was 0.027 g/cm^2^ for L1–L4 (1.1%); 0.034 g/cm^2^ for the femoral neck (2.0%) and 0.032 g/cm^2^ (1.6%) for the total hip.

Physical activity (PA) was defined using the WHO concept (available at http://www.who.int/topics/physical_activity/en/) as any bodily movement produced by skeletal muscles that requires energy expenditure, and it was qualitatively evaluated as yes or no. The calcium daily intake was estimated by the sum of the dietary and supplemental ingestion.

The safety and tolerability were recorded at each visit by the investigators. Major hypoglycemia was defined as an event that required the assistance of another individual to actively administer glucagon, carbohydrate, or other resuscitative measures. Minor hypoglycemia was defined as an event during which typical symptoms of hypoglycemia were accompanied by a measured plasma glucose ≤70 mg/dL (3.9 mmol/L) or not accompanied by classic symptoms of hypoglycemia but with a measured plasma glucose ≤70 mg/dL. An event during which symptoms of hypoglycemia were not accompanied by a plasma glucose determination, but that was presumably caused by a plasma glucose ≤70 mg/dL was considered minor hypoglycemia.

### Statistical analysis

The sample size was calculated to obtain a significant result in at least one of the primary outcomes (difference >30% in bone remodeling markers). The change in the OC marker was applied for the calculation. Nineteen patients per group were required to provide 80% power and a 0.05% significance level to detect a difference between means of at least 30% among the two treatments. The expected screening failure rate was 30%, and the estimated dropout rate was 8%; thus, 56 patients were planned for enrollment.

The baseline characteristics were summarized based on the dataset of the intention to treat (ITT) population. The key features at baseline were represented, and comparisons among groups were made using Student *t* tests (parametric variables with normal distribution) or Mann–Whitney tests (non-normal distribution). For primary and secondary variables, which were all quantitative and of a normal distribution, Student *t* tests were used for the statistical analysis. The final statistical analysis of the results was performed taking into consideration only the difference between the means of the analyzed parameters in the 6th and 12th months and the baseline value. Statistical analysis was performed using SPSS for Windows (version 20.0; SPSS, Chicago, IL, USA). All inferential statistical tests were conducted at a p < 0.05 (two-sided). Unless otherwise stated, data are presented as the mean ± standard deviation (SD).

## Results

**Fig. 1:**
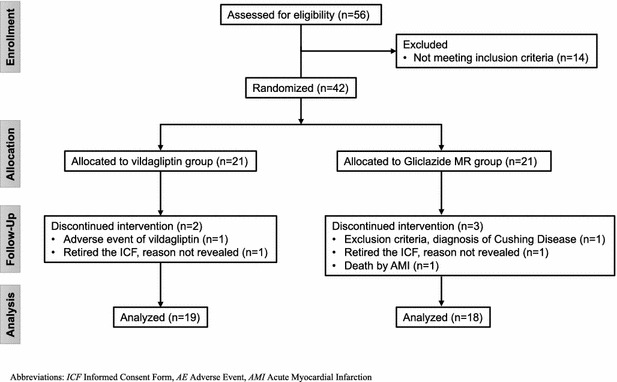
Patient flow chart. *ICF* informed consent form, *AE* adverse event, *AMI* acute myocardial infarction

Fifty-six patients were enrolled between October 2012 and October 2014. There were 14 screening failures. The remaining women were randomly assigned in a 1:1 manner to vildagliptin (n = 21) or gliclazide MR (n = 21) groups, both in addition to their usual treatment for T2D. Five subjects did not complete the study procedures; thus, 37 patients completed the 12-month follow-up, including 19 patients in the vildagliptin group and 18 patients in the gliclazide MR group (Fig. 1). The trial ended when the last subject included completed the 12-month follow-up.

The baseline clinical characteristics were not statistically different between the treatment groups (Table 1). The physical activity status remained stable throughout the 12-month period in all individuals.

**Table 1:** Baseline characteristics and patient disposition

	Vildagliptin (N = 21)	Gliclazide MR (N = 21)	p value
Age (years)	62.8 ± 6.4	61 ± 5.3	0.324
Time since menopause (years)	14 ± 8.5	13.1 ± 8.1	0.740
Race, n (%)			
Caucasian	18 (85.7)	19 (90.4)	NA
Black	2 (9.5)	1(4.8)	NA
Hispanic or latino	1 (4.8)	1 (4.8)	NA
Physical activity, n (%)	9 (42.9)	9 (42.9)	1.000
Calcium intake (mg/day)^a^	811 ± 403	824 ± 397	0.787
Metformin daily dose (mg)	1640.5 ± 664.2	1742.9 ± 626.4	0.610
Body weight (kg)	77.3 ± 15.7	82.7 ± 20.1	0.336
BMI (kg/m^2^)	31.7 ± 5.8	33.8 ± 6.7	0.287
Serum calcium (mg/dL)	9.3 ± 0.4	9.3 ± 0.4	0.661
Serum phosphorous (mg/dL)	3.7 ± 0.4	3.8 ± 0.5	0.512
Serum creatinine (mg/dL)	0.78 ± 0.15	0.75 ± 0.11	0.483
Vitamin D (25OH) (mg/dL)	34.7 ± 10.1	34.9 ± 19.8	0.963
Calcitonin (pg/mL)^b^	<2.0	<2.0	NA
AST (U/L)	23.6 ± 9.3	30.0 ± 13.5	0.080
ALT (U/L)	29.5 ± 19.2	34.8 ± 19.2	0.373
FPG (mg/dL)	144.0 ± 22.4	146.8 ± 28.5	0.729
PPG (mg/dL)	159.4 ± 48.6	147.1 ± 54.6	0.446
HbA1c (%)/(mmol/mol)	7.35 ± 0.54/57 ± 5.9	7.32 ± 0.60/56 ± 6.6	0.851

Variables are presented as the mean ± SD

*BMI* body mass index, *AST* aspartate aminotransferase, *ALT* alanine aminotransferase, *FPG* fasting plasma glucose, *PPG* 2-h postprandial plasma glucose, *NA* not applicable

^a^ Both dietary and supplementation

^b^ Lower limit of detection of the method is 2 pg/mL

The vildagliptin dose was 100 mg/day (50 mg in two daily intakes) for all patients. The gliclazide MR dose achieved was 72.6 ± 21.1 mg in the 6th month and 73.3 ± 25.7 in the 12th month.

The results of the 6 and 12 months of treatment with vildagliptin or gliclazide MR with respect to the investigated variables are listed in Table 2. For the primary variables, which include the levels of bone turnover markers, there was no significant difference between the vildagliptin and gliclazide MR groups when the differences between the values obtained at months 6 and 12 were compared with the baseline.

**Table 2:** Baseline values and changes in bone remodeling marker values in vildagliptin and gliclazide MR treatment groups

Variables	Vildagliptin (N = 21)	Gliclazide MR (N = 21)	*p* value (among groups)
OC (ng/mL)			
Baseline	12.5 ± 4.2	10.2 ± 3.5	0.063
Change from baseline to month 6	1.0 ± 3.5	0.5 ± 2.9	0.622
*p* value*	0.204	0.438	
Change from baseline to month 12	−0.1 ± 2.9	0.6 ± 3.8	0.519
*p* value*	0.930	0.468	
PINP (ng/mL)			
Baseline	38.1 ± 11.8	36.3 ± 14.7	0.660
Change from baseline to month 6	2.4 ± 14.9	−0.3 ± 8.3	0.475
*p* value*	0.462	0.887	
CTX (ng/mL)			
Baseline	0.288 ± 0.139	0.197 ± 0.062	0.013
Change from baseline to month 6	0.010 ± 0.071	0.029 ± 0.052	0.355
*p* value*	0.523	0.023	
Change from baseline to month 12	0.001 ± 0.153	0.008 ± 0.060	0.858
*p* value*	0.563	0.972	
U-NTX (nmol/BCE/mmol creatinine)			
Baseline	41.1 ± 16.5	35.6 ± 14.4	0.255
Change from baseline to month 6	8.1 ± 25.9	−2.2 ± 16.4	0.138
*p* value*	0.167	0.555	
Change from baseline to month 12	−1.1 ± 25.8	−1.9 ± 19.3	0.902
*p* value*	0.854	0.660	

Variables are expressed as the mean ± SD

*OC* osteocalcin, *PINP* amino-terminal propeptide of procollagen type 1, *CTX* carboxy-terminal telopeptide of type 1 collagen, *U*-*NTX* urinary amino-terminal telopeptide of type 1 collagen

* *p* value after therapy versus baseline

**Fig. 2:**
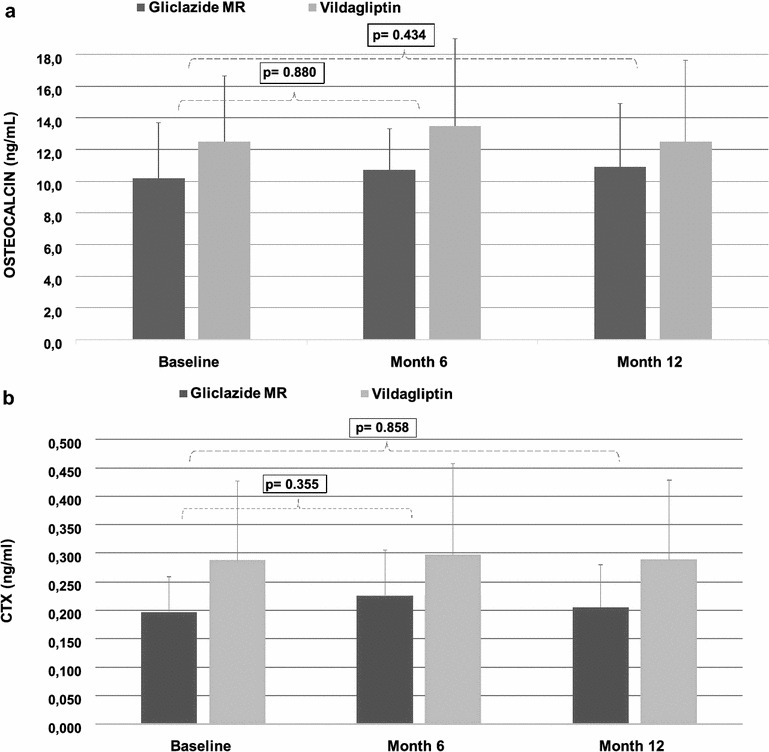
Progression of osteocalcin (**a**) and CTX (**b**) serum levels during the 12-month period (*p* values calculated for differences between means in months 6 and 12 versus baseline)

After 6-month treatment, the OC was 13.5 ± 5.5 in the vildagliptin group versus 10.7 ± 2.6 ng/mL in the gliclazide MR group. At the end of treatment (month 12), the values were 12.5 ± 5.2 versus 10.9 ± 4.0 ng/mL, respectively. An analysis of the differences between months 6 and 12 versus baseline indicates that there is no significant difference in the effect of the drugs on this marker (p = 0.623 and p = 0.519 for months 6 and 12, respectively) (Fig. 2a).

The bone formation marker PINP was analyzed only at baseline and month 6. In month 6, the serum PINP dosage was 41.8 ± 20.6 in the vildagliptin group versus 35.6 ± 15.7 ng/mL in the gliclazide MR group. As shown in Table 2, there was no difference between both medications in their effect on PINP (p = 0.661) for the difference between month 6 and baseline.

At baseline, the CTX values were higher in the vildagliptin group (0.288 ± 0.139 versus 0.197 ± 0.062 ng/mL in the gliclazide MR group, p = 0.013). In month 6, CTX was 0.258 ± 0.159 versus 0.226 ± 0.080 ng/mL, respectively, and, at the 12th month, the CTX values were 0.289 ± 0.140 versus 0.205 ± 0.074 ng/mL, respectively. Despite the significant difference between the groups in the baseline, there was no difference in the effect of the drugs on this marker when we analyzed only the differences between the means in months 6 and 12 versus baseline (p = 0.355 and p = 0.858 for the 6th and 12th months, respectively) (Fig. 2b).

After a 6-month treatment, the U-NTX levels were 49.2 ± 26.8 in the vildagliptin group versus 33.4 ± 9.8 nmol BCE/mmol creatinine in the gliclazide MR group. At the end of treatment (month 12), the values were 40.1 ± 23.0 versus 33.6 ± 15.2 nmol BCE/mmol creatinine, respectively. There was a significant difference between the groups in the 6th month; however, when the differences between means in months 6 and 12 and baseline were analyzed, there is no significant difference in the effect of the drugs on this marker (p = 0.138 and p = 0.902 for months 6 and 12, respectively).

The secondary variables, including the BMD of the lumbar spine, femoral neck and total hip, did not exhibit significant differences between groups at baseline or month 12 or the difference between the end of the treatment and baseline (Table 3).

**Table 3:** Bone mineral density (BMD) of the lumbar spine, femoral neck and total hip

	Vildagliptin N = 19		Gliclazide MR N = 18		*p* value
	Mean	Standard deviation	Mean	Standard deviation	
BMD of the lumbar spine (g/cm^2^)					
Baseline	0.956	0.212	0.972	0.121	0.771
Month 12	0.956	0.211	0.964	0.118	0.880
Difference from baseline	0.000	0.025	−0.008	0.036	0.434
BMD of the femoral neck (g/cm^2^)					
Baseline	0.777	0.161	0.791	0.092	0.747
Month 12	0.781	0.172	0.801	0.116	0.659
Difference from baseline	0.004	0.055	0.011	0.033	0.612
BMD of the total hip (g/cm^2^)					
Baseline	0.923	0.144	0.934	0.108	0.789
Month 12	0.921	0.154	0.935	0.113	0.743
Difference from baseline	−0.002	0.029	0.002	0.023	0.648

The fasting glucose reduced 12.5 ± 34.0 mg/dL in month 12 compared with the baseline value in the vildagliptin group. The variation was 31.1 ± 34.6 mg/dL in the gliclazide MR group, and the difference between the groups from the baseline was not significant (p = 0.086).

The postprandial glucose values presented a reduction of 11.1 ± 64.9 mg/dL in month 12 compared with the baseline in the vildagliptin group. The decrease was 28.9 ± 58.0 in the gliclazide MR group (p = 0.354).

HbA1c exhibited a reduction of 0.39 ± 0.92 percentage point in the vildagliptin group versus 0.80 ± 0.92 in the gliclazide MR group when month 12 was compared with the baseline (p = 0.354). The glucose and safety parameters are available in the Additional file 1.

## Discussion

Our study is the first investigation to show that 1 year of vildagliptin treatment has the same neutral effect as a sulfonylurea on BMD and markers of bone metabolism in postmenopausal women with T2D. Bone safety is currently an extremely relevant topic, and the present study suggests that sulfonylureas and incretin-based therapies do not appear to have an adverse impact on bone metabolism issues as thiazolidinediones or canagliflozin [16–23].

Both bone formation (PINP and osteocalcin) and bone resorption markers (CTX and U-NTX) did not exhibit significant changes over the follow-up period compared with the baseline in both groups, which suggests that both drugs have similar bone metabolism safety profiles. The BMD values corroborate with these findings, as none of the groups gained or lost bone density after 12 months of treatment. Considering that sulfonylureas likely have the lowest impact on bone metabolism among the oral antidiabetics [23] and are not associated with changes in the risk of fracture [2], the results of this study suggest that the iDPP-4 vildagliptin has a similar bone metabolism safety profile and presumably does not increase the risk of fracture, as the sulfonylurea gliclazide. Liraglutide, a GLP-1 receptor agonist with similar features as the DPP-4 inhibitors, has previously demonstrated a similar BMD pattern to a sulfonylurea [24].

Preclinical studies have indicated that the incretin hormones GLP-2 and GIP play a significant role in bone metabolism via the stimulation of bone formation and inhibition of bone resorption [4–10]. Inhibition of the DPP-4 enzyme increases the levels of both GLP-2 and GIP, which suggests that the class of DPP-4 inhibitors, in addition to controlling glucose levels, may positively affect the bone metabolism. The results in rodents are controversial regarding iDDP-4. Sitagliptin demonstrated neutral results concerning the thickness of the cortical bone in ovariectomized mice and an improvement in BMD and trabecular architecture in a comparative study with pioglitazone [11, 25].

Most studies in humans have demonstrated that the iDDP-4 class has at least a neutral effect on bone metabolism and the risk of fractures [26–28]. A meta-analysis with 11,880 iDDP-4 users suggested a reduction of up to 40% in the risk of fracture [12]. In contrast, a retrospective population-based cohort study (N = 216,816) demonstrated no different risk of fracture when iDPP-4 users were compared with controls [26]. Furthermore, a more recent meta-analysis of 62 randomized clinical trials (N = 62,206 patients) demonstrated that iDPP-4 does not affect the risk of fracture compared with placebo or other antidiabetic medications [29]. None of these meta-analyses or further studies directly examined the effect of vildagliptin with a sulfonylurea. Moreover, no randomized clinical trial has compared the bone effects of an iDPP-4 with the sulfonylurea gliclazide MR, the only drug in the class that is suggested to have a good cardiovascular safety profile [14]. The few real comparisons between iDPP-4 and sulfonylureas are derived from studies in which fractures were reported as adverse events and the comparison was made with glipizide or glimepiride.^1^

The current study corroborates most previous studies, with the difference in the comparison of an iDDP-4 to an oral antidiabetic class established to be safe in a prospective, randomized, 12-month duration study. Another strength of our study is that it was performed in a population in which fractures and changes in bone metabolism are more evident, namely, postmenopausal women. The presence of T2D for more than 7 years further increases this risk. Moreover, no fracture was identified in any patient during the 12-month treatment.

The study has several limitations that must be recognized. One limitation is the sample size, which was calculated based on the osteocalcin values for postmenopausal women with T2D [30]. A greater number of individuals analyzed may demonstrate a difference between the treatments. A longer follow-up may also indicate differences in the BMD not identified in our study with 12 months of active treatment. The presence of a previous fragility fracture was one of the exclusion criteria of the study; unfortunately, there are no data on spine X-ray to assess vertebral fracture. Moreover, physical activity was only qualitatively evaluated. An evaluation of the daily physical activity, expressed as metabolic equivalents (METs), would have provided more valuable information.

Recently, an interim result of a phase IV trial demonstrated that canagliflozin, a sodium-glucose transporter-2 inhibitor (iSGLT2), may be associated with an increased risk of atypical fractures [31]. Thiazolidinediones are a classical example that an antidiabetic treatment may negatively affect bone and increase the fracture risk [16–18]. Taking this finding into consideration, anti-diabetic drugs, such as iDPP4, that do not cause further harm to bone are reassuring. Longer studies that include a greater number of patients are necessary to demonstrate, in a conclusive manner, iDPP-4 effects on bone metabolism and BMD.

## Conclusions

Bone turnover markers and BMD remained unchanged after a 12-month treatment in both groups, which suggests that vildagliptin has the same safety profile as gliclazide MR on bone metabolism.

## Additional file

**Additional file 1: Table S1.** Baseline values and changes in glucose and safety parameters in vildagliptin and gliclazide MR treatments. **Table S2.** Summary and analysis of non-serious adverse events during the study period, divided by treatment group.

## Abbreviations

25-OH-vit.D: 25-hidroxi-vitamin D
ALT: alanine aminotransferase
AST: aspartate aminotransferase
BMD: bone mineral density
BMI: body mass index
BTM: bone turnover markers
CTX: carboxy-terminal telopeptide of type 1 collagen
DXA: dual-energy X-ray absorptiometry
FPG: fasting plasma glucose
GH: growth hormone
GIP: glucose-dependent insulinotropic peptide
GLP-2: glucagon-like peptide-2
HbA1c: glycated hemoglobin
HPLC: high-pressure liquid chromatography
iDPP-4: dipeptidyl peptidase-4 inhibitors
IGF-1: insulin-like growth factor 1
ISCD: International Society for Clinical Densitometry
iSGLT2: sodium–glucose transporter-2 inhibitor
ITT: intention to treat
MR: modified release
OC: osteocalcin
PINP: amino-terminal propeptide of procollagen type I
PPG: postprandial glucose
PTH: parathyroid hormone
SD: standard deviation
T2D: type 2 diabetes
U-NTX: urinary amino-terminal telopeptide of type 1 collagen

## Footnotes

1 Data not published. Available from https://clinicaltrials.gov (NCT00856284, NCT00707993, NCT01006603, NCT00575588, NCT00509236, NCT00509262, NCT00701090, NCT00086515, and NCT00094770).

## References

[1] Farr JN, Drake MT, Amin S, Ill JM, McCready LK, Khosla S (2014). In vivo assessment of bone quality in postmenopausal women with type 2 diabetes. J Bone Miner Res.

[2] Lecka-Czernik B (2013). Safety of anti-diabetic therapies on boné. Clin Rev Bone Miner Metab..

[3] Moreira CA, Barreto FC, Dempster DW (2015). New insights on diabetes and bone metabolism. J Bras Nefrol.

[4] Dicembrini I, Mannucci E, Rotella CM (2012). Bone: incretin hormones perceiver or receiver?. Exp Diabetes Res.

[5] Clowes JA, Khosla S, Eastell R (2005). Potential role of pancreatic and enteric hormones in regulating bone turnover. J Bone Miner Res.

[6] Tsukiyama K, Yamada Y, Yamada C, Harada N, Kawasaki Y, Ogura M, Bessho K, Li M, Amizuka N, Sato M, Udagawa N, Takahashi N, Tanaka K, Oiso Y, Seino Y (2006). Gastric inhibitory polypeptide as an endogenous factor promoting new bone formation after food ingestion. Mol Endocrinol.

[7] Zhong Q, Itokawa T, Sridhar S, Ding KH, Xie D, Kang B, Bollag WB, Bollag RJ, Hamrick M, Insogna K, Isales CM (2007). Effects of glucose-dependent insulinotropic peptide on osteoclast function. Am J Physiol Endocrinol Metab.

[8] Xie D, Cheng H, Hamrick M, Zhong Q, Ding KH, Correa D, Williams S, Mulloy A, Bollag W, Bollag RJ, Runner RR, McPherson JC, Insogna K, Isales CM (2005). Glucose-dependent insulinotropic polypeptide receptor knockout mice have altered bone turnover. Bone.

[9] Drucker DJ, Shi Q, Crivici A, Sumner-Smith M, Tavares W, Hill M, DeForest L, Cooper S, Brubaker PL (1997). Regulation of the biological activity of glucagon-like peptide 2 in vivo by dipeptidyl peptidase IV. Nat Biotechnol.

[10] Henriksen DB, Alexandersen P, Bjarnason NH, Vilsbøll T, Hartmann B, Henriksen EE, Byrjalsen I, Krarup T, Holst JJ, Christiansen C (2003). Role of gastrointestinal hormones in postprandial reduction of bone resorption. J Bone Miner Res.

[11] Kyle KA, Willett TL, Baggio LL, Drucker DJ, Grynpas MD (2011). Differential effects of PPAR-gamma activation versus chemical or genetic reduction of DPP-4 activity on bone quality in mice. Endocrinology.

[12] Monami M, Dicembrini I, Antenore A, Mannucci E (2011). Dipeptidyl peptidase-4 inhibitors and bone fractures: a meta-analysis of randomized clinical trials. Diabetes Care.

[13] Al Sifri S, Basiounny A, Echtay A, Al Omari M, Harman-Boehm I, Kaddaha G, Al Tayeb K, Mahfouz AS, Al Elq A, Radican L, Ozesen C, Katzeff HL, Musser BJ, Suryawanshi S, Girman CJ, Davies MJ, Engel SS, 2010 Ramadan Study Group (2011). The incidence of hypoglycaemia in Muslim patients with type 2 diabetes treated with sitagliptin or a sulphonylurea during Ramadan: a randomised trial. Int J Clin Pract.

[14] Patel A, MacMahon S, Chalmers J, Neal B, Billot L, Woodward M, Marre M, Cooper M, Glasziou P, Grobbee D, Hamet P, Harrap S, Heller S, Liu L, Mancia G, Mogensen CE, Pan C, Poulter N, Rodgers A, Williams B, Bompoint S, de Galan BE, Joshi R, Travert F, ADVANCE Collaborative Group (2008). Intensive blood glucose control and vascular outcomes in patients with type 2 diabetes. N Engl J Med.

[15] Urbaniak GC, Plous S. Research Randomizer (Version 4.0). 2013. http://www.randomizer.org/. Accessed 22 Jun 2013.

[16] Rejnmark KL (2008). Bone effects of glitazones and other anti-diabetic drugs. Curr Drug Saf..

[17] Dormuth CR, Carney G, Carleton B, Bassett K, Wright JM (2009). Thiazolidinediones and fractures in men and women. Arch Intern Med.

[18] Grey A (2008). Skeletal consequences of thiazolidinedione therapy. Osteoporos Int.

[19] Kahn SE, Haffner SM, Heise MA, Herman WH, Holman RR, Jones NP, Kravitz BG, Lachin JM, O’Neill MC, Zinman B, Viberti G, ADOPT Study Group (2006). Glycemic durability of rosiglitazone, metformin, or glyburide monotherapy. N Engl J Med.

[20] Grey A, Bolland M, Gamble G, Wattie D, Horne A, Davidson J, Reid IR (2007). The peroxisome proliferator-activated receptor-gamma agonist rosiglitazone decreases bone formation and bone mineral density in healthy postmenopausal women: a randomized, controlled trial. J Clin Endocrinol Metab.

[21] Thrailkill KM, Clay Bunn R, Nyman JS, Rettiganti MR, Cockrell GE, Wahl EC, Uppuganti S, Lumpkin CK, Fowlkes JL (2016). SGLT2 inhibitor therapy improves blood glucose but does not prevent diabetic bone disease in diabetic DBA/2J male mice. Bone.

[22] Bilezikian JP, Watts NB, Usiskin K, Polidori D, Fung A, Sullivan D, Rosenthal N (2016). Evaluation of bone mineral density and bone biomarkers in patients with type 2 diabetes treated with canagliflozin. J Clin Endocrinol Metab.

[23] Zinman B, Haffner SM, Herman WH, Holman RR, Lachin JM, Kravitz BG, Paul G, Jones NP, Aftring RP, Viberti G, Kahn SE, ADOPT Study Group (2010). Effect of rosiglitazone, metformin, and glyburide on bone biomarkers in patients with type 2 diabetes. J Clin Endocrinol Metab.

[24] Gilbert MP, Marre M, Holst JJ, Garber A, Baeres FM, Thomsen H, Pratley RE (2016). Comparison of the long-term effects of liraglutide and glimepiride monotherapy on bone mineral density in patients with type 2 diabetes. Endocr Pract.

[25] Cusick T, Mu J, Pennypacker BL, Li Z, Scott KR, Shen X, Fisher JE, Langdon RB, Kimmel DB, Zhang BB, Glantschnig H (2013). Bone loss in the oestrogen-depleted rat is not exacerbated by sitagliptin, either alone or in combination with a thiazolidinedione. Diabetes Obes Metab.

[26] Driessen JH, van Onzenoort HA, Henry RM, Lalmohamed A, van den Bergh JP, Neef C, Leufkens HG, de Vries F (2014). Use of dipeptidyl peptidase-4 inhibitors for type 2 diabetes mellitus and risk of fracture. Bone.

[27] Majumdar SR, Josse RG, Lin M, Eurich DT (2016). Does sitagliptin affect the rate of osteoporotic fractures in type 2 diabetes? Population-based cohort study. J Clin Endocrinol Metab.

[28] Bunck MC, Poelma M, Eekhoff EM, Schweizer A, Heine RJ, Nijpels G, Foley JE, Diamant M (2012). Effects of vildagliptin on postprandial markers of bone resorption and calcium homeostasis in recently diagnosed, well-controlled type 2 diabetes patients. J Diabetes.

[29] Fu J, Zhu J, Hao Y, Guo C, Zhou Z (2016). Dipeptidyl peptidase-4 inhibitors and fracture risk: an updated meta-analysis of randomized clinical trials. Sci Rep.

[30] Kanazawa I, Yamaguchi T, Yamamoto M, Yamauchi M, Kurioka S, Yano S, Sugimoto T (2009). Serum osteocalcin level is associated with glucose metabolism and atherosclerosis parameters in type 2 diabetes mellitus. J Clin Endocrinol Metab.

[31] Watts NB, Bilezikian JP, Usiskin K, Edwards R, Desai M, Law G, Meininger G (2016). Effects of canagliflozin on fracture risk in patients with type 2 diabetes mellitus. J Clin Endocrinol Metab.

