# Supplementary material for: Vildagliptin has the same safety profile as a sulfonylurea on bone metabolism and bone mineral density in post-menopausal women with type 2 diabetes: a randomized controlled trial
Source: Diabetol Metab Syndr. 2017 May 15;9:35. doi: 10.1186/s13098-017-0232-2 (PMC5433032; doi:10.1186/s13098-017-0232-2)
Supplement: Supplementary file 1 — Additional file 1: Table S1. Baseline values and changes in glucose and safety parameters in vildagliptin and gliclazide MR treatments. Table S2. Summary and analysis of non-serious adverse events during the study period, divided by treatment group. [file 13098_2017_232_MOESM1_ESM.docx]

- **Vildagliptin has the same safety profile as a sulfonylurea on bone metabolism and bone mineral density in post-menopausal women with Type 2 Diabetes: a randomized controlled trial.**
- Vianna AGD^a,b^, Lacerda CS^b,c^, Pechmann LM^b^, Polesel MG^b^, Marino EC^b^, Borba VZC^c^, Barreto FC^d^
- **Authors’ full names in order of appearance:**
- Andre Gustavo Daher Vianna, M.D.^a,b^ drandrevianna@gmail.com
- Claudio Silva de Lacerda, M.D., MSc.^b,c^ claudiodelacerda@yahoo.com.br
- Luciana Muniz Pechmann, M.D.^b^ lucianapechmann@gmail.com
- Michelle Garcia Polesel, M.D.^b^ mipolesel@hotmail.com
- Emerson Cestari Marino, M.D.^b^ ememarino@hotmail.com
- Victoria Zeghbi Cochenski Borba, M.D., Ph.D.^c^ vzcborba@gmail.com
- Fellype de Carvalho Barreto, M.D., Ph.D.^d^ fellype.barreto@terra.com.br
- **Affiliations:**
- a. Pontifical Catholic University of Parana, Curitiba, Brazil.
- Rua Imaculada Conceição, 1155 – Bloco Medicina – Prado Velho
- Curitiba, Parana, Brazil
- ZIP Code: 80215-901
- E-mail: ppgcs@pucpr.br
- b. Curitiba Diabetes Center, Division of Endocrinology, Hospital Nossa Senhora das Graças, Curitiba, Brazil.
- Rua Alcides Munhoz, 433 – 4º andar - Mercês
- Curitiba, Paraná, Brazil
- ZIP Code: 80810-040
- Phone number: + 55 41 3023 12 52
- Fax number: + 55 41 3079 86 33
- E-mail: [adm@centrodediabetescuritiba.com.br](mailto:adm@centrodediabetescuritiba.com.br)
- c. Division of Endocrinology, Department of Internal Medicine, Federal University of Paraná, Curitiba, Brazil.
- Avenida Agostinho Leão Junior, 285 – Alto da Gloria
- Curitiba, Paraná, Brazil
- ZIP Code: 80030-110
- E-mail: cesarluiz@hc.ufpr.br
- d. Division of Nephrology, Department of Internal Medicine, Federal University of Paraná, Curitiba, Brazil.
- Rua General Carneiro,181 – Alto da Gloria
- Curitiba, Paraná, Brazil
- ZIP Code 80060-900
- **Corresponding Author:** André Gustavo Daher Vianna
- Centro de Diabetes Curitiba
- Rua Alcides Munhoz, 433, Curitiba, Paraná, Brazil
- ZIP Code: 80810-040
- Phone number: + 55 41 3023 12 52
- Fax number: + 55 41 3079 86 33
- E-mail: [drandrevianna@gmail.com](mailto:drandrevianna@gmail.com)
- ORCID ID: 0000-0002-0497-5062
- Registered at Clinical Trials.gov with number NCT01679899
- **SUPPLEMENTARY MATERIAL**

The safety and glucose parameters are detailed in Table S1. The levels of AST, ALT, and calcitonin, secondary safety variables, did not exhibit a significant change compared with the baseline in both groups in month 6, and significant reductions of the AST and ALT levels were identified in the gliclazide MR group in month 12. The calcitonin value was below the limit of detection in all patients (<2 pg/mL) at baseline and month 12. There was one single exception, at baseline, in which one patient presented calcitonin equal to 2.3 pg/mL, a value that is equally considered low and within normal limits.

**Table S1** Baseline values and changes in glucose and safety parameters in vildagliptin and gliclazide MR treatments

| **Characteristics** | **Vildagliptin**  **(N=21)** | **Gliclazide MR**  **(N=21)** | ***P*-value**  **(among groups)** |
| --- | --- | --- | --- |
| FPG (mg/dL) |  |  |  |
| Baseline | 144.0 ± 22.4 | 146.8 ± 28.5 | 0.729 |
| Change from baseline | -13.2 ± 32.4 | -24.2 ± 37.9 | 0.318 |
| to month 6 |  |  |  |
| *p* value* | 0.077 | 0.008 |  |
| Change from baseline | -12.5 ± 34.0 | -31.1 ± 34.6 | 0.086 |
| to month 12 |  |  |  |
| *p* value* | 0.107 | 0.001 |  |
| PPG (mg/dL) |  |  |  |
| Baseline | 159.4 ± 48.6 | 147.1 ± 54.6 | 0.446 |
| Change from baseline | -13.0 ± 45.2 | -17.2 ± 74.1 | 0.826 |
| to month 6 |  |  |  |
| *p* value* | 0.201 | 0.299 |  |
| Change from baseline | -11.1 ± 64.9 | -28.9 ± 58.0 | 0.354 |
| to month 12 |  |  |  |
| *p* value* | 0.443 | 0.033 |  |
| HbA1c (%) (mmol/mol) |  |  |  |
| Baseline | 7.35 ± 0.54 (57 ± 5.9) | 7.32 ± 0.60 (56 ± 6.6) | 0.851 |
| Change from baseline |  |  |  |
| to month 6 | -0.51 ± 0.77 (-5.6 ± 8.4) | -0.74 ± 0.92 (-8.1 ± 10.1) | 0.376 |
| *p* value* | 0.007 | 0.001 |  |
| Change from baseline | -0.39 ± 0.92 (-4.3 ± 10.1) | -0.80 ± 0.92 (- 8.7 ± 10.1) | 0.151 |
| to month 12 |  |  |  |
| *p* value* | 0.068 | 0.001 |  |
| AST (U/L) |  |  |  |
| Baseline | 23.6 ± 9.3 | 30.0 ± 13.5 | 0.120 |
| Change from baseline | 0.5 ± 5.3 | -2.1 ± 8.9 | 0.230 |
| to month 6 |  |  |  |
| *p* value* | 0.586 | 0.368 |  |
| Change from baseline | -0.2 ± 6.7 | -4.0 ± 9.9 | 0.176 |
| to month 12 |  |  |  |
| *p* value* | 0.723 | 0.046 |  |
| ALT (U/L) | 29.5 ± 19.2 | 34.8 ± 19.2 | 0.330 |
| Baseline |  |  |  |
| Change from baseline | -0.9 ± 6.7 | -2.1 ± 12.1 | 0.960 |
| to month 6 |  |  |  |
| *p* value* | 0.508 | 0.573 |  |
| Change from baseline | -0.5 ± 7.9 | -4.7 ± 10.7 | 0.114 |
| to month 12 |  |  |  |
| *p* value* | 0.811 | 0.022 |  |
| Calcitonin (pg/mL) |  |  |  |
| Baseline | <2.0 | <2.0 | NA |
| Month 12 | <2.0 | <2.0 | NA |
| *p* value* | NA | NA |  |

Variables are expressed as mean ± SD, *FPG* fasting plasma glucose, *PPG* postprandial glucose, *HbA1c* glycated hemoglobin, *AST* aspartate aminotransferase, *ALT* alanine aminotransferase, *NA* not applicable, **p*-value after therapy versus baseline

Vildagliptin and gliclazide MR treatments were largely well tolerated. Adverse events are presented in Table S2. No bone fractures were reported during the follow-up period. There was no major hypoglycemia; seven events of minor hypoglycemia were reported in the gliclazide MR group compared with 2 events in the vildagliptin group (p= 0.062). Urinary tract infection (UTI) occurred in 3 cases in the vildagliptin group versus no case in the gliclazide MR group (p=0.230). There were two serious adverse events (SAEs) reported during the conduction of the study, including a hemorrhagic stroke in the vildagliptin group and death as a result of an acute myocardial infarction in the gliclazide MR group. The SAEs were not likely related to the study medication and were properly informed to pharmacovigilance and regulatory and ethical authorities.

**Table S2.** Summary and analysis of non-serious adverse events during the study period, divided by treatment group

|  | **Vildagliptin** | **Gliclazide MR** | ***P* value*** |
| --- | --- | --- | --- |
|  | **(N = 21)** | **(N= 21)** |  |
| Stable angina | 1 | 0 | 1 |
| Arthrosis in the lower limbs | 0 | 1 | 0.486 |
| Asthenia, fatigue | 2 | 1 | 1 |
| Burning feet | 1 | 0 | 1 |
| Migraine | 1 | 0 | 1 |
| Cystitis (UTI) and dysuria | 3 | 0 | 0.230 |
| Depression | 0 | 1 | 0.486 |
| Diarrhea | 1 | 0 | 1 |
| Dyspepsia | 0 | 2 | 0.230 |
| Abdominal pain | 2 | 0 | 0.486 |
| Muscle and limb pain | 2 | 1 | 1 |
| Edema in limbs | 2 | 1 | 1 |
| Epicondylitis in elbow | 1 | 0 | 1 |
| Gastritis | 1 | 0 | 1 |
| Hemorrhoids | 0 | 1 | 0.486 |
| Hypertriglyceridemia | 1 | 0 | 1 |
| Hypoglycemia | 2 | 7 | 0.062 |
| Hypothyroidism | 1 | 0 | 1 |
| Upper respiratory infection | 1 | 0 | 1 |
| Labyrinthopathy | 0 | 1 | 0.486 |
| Nausea | 2 | 1 | 1 |
| Papillary nevus | 1 | 0 | 1 |
| Constipation | 0 | 1 | 0.486 |
| Paresthesia in limbs | 2 | 0 | 0.486 |
| Itching eye | 1 | 0 | 1 |
| Sweating | 1 | 0 | 1 |
| Tachycardia | 1 | 0 | 1 |
| Dizziness | 1 | 0 | 1 |
| Cough | 1 | 0 | 1 |
| Tremors | 0 | 2 | 0.230 |
| Urticaria | 1 | 0 | 1 |
| **Total non-serious adverse events** | **33** | **20** |  |

The occurrence of adverse events is represented by absolute numbers. *UTI* urinary tract infection.

**p*-value was obtained by the Fisher’s exact test.
